# A cooling cuff compared to a moist ice pack on radial artery blood flow and lumen diameter in healthy participants

**DOI:** 10.4102/hsag.v23i0.1040

**Published:** 2018-09-20

**Authors:** Joshua Gernetzky, Laura O’Connor, Desiree Varatharajullu, Zombuso C. Dludla

**Affiliations:** 1 Department of Chiropractic and Somatology, Durban University of Technology, South Africa; 2 Department of Radiography, Durban University of Technology, South Africa

## Abstract

**Background:**

Cryotherapy is a favourable treatment for post-traumatic injuries in the acute stage because of its effect on inflammation and pain. A novel cooling cuff, which can be easily used and can be wrapped around the injured area that does not require freezing, has been developed. Its efficacy compared to traditional ice therapy has not been established.

**Aim:**

To establish the effect of a cooling cuff on radial artery blood flow and lumen diameter compared to moist ice.

**Setting:**

Chiropractic clinic and radiographic laboratory.

**Method:**

A controlled laboratory pre-test post-test investigation assessed asymptomatic participants who were randomly allocated into a moist ice pack (*n* = 22) or the cooling cuff (*n* = 21) group. The intervention was placed on the participants forearm over the radial artery for 15 min. Data was collected by a qualified diagnostic radiographer using Doppler ultrasound. Data was analysed, using repeated measures analysis of variance to assess changes in blood flow and lumen diameter pre- and post-intervention. A *p*-value of less than 0.05 was considered significant.

**Results:**

Both groups showed a significant decrease in radial artery blood flow (*p* < 0.001) after 15 min with no significant changes being observed in diameter size. No significant differences were observed between the groups for radial artery blood flow or diameter.

**Conclusion:**

The cooling cuff resulted in a similar effect on radial artery blood flow and lumen diameter as moist ice, indicating that patients and practitioners may utilise the cooling cuff in the acute phases of an injury to alter blood flow.

## Introduction

### Background to the study

Cryotherapy techniques are commonly used as the first line of treatment for numerous types of post-traumatic injuries (Smith et al. 1993), as they may increase pain threshold and reduce inflammation when applied within days of an injury (Bleakley, McDonough & Auley 2004). There are many different cryotherapy techniques (Cameron 2013; Michlovitz 1996) used by professionals such as medical doctors, manual therapists like physiotherapists and chiropractors, and by sports physicians (Enwemka et al. 2002). Non-professionals also use this therapeutic option regularly to treat acute musculoskeletal injuries like low-back and neck pain as well as sports injuries such as sprains, strains and contusions (Michlovitz 1996; Nadler, Weingand & Kruse 2004). Common methods include ice and gel packs, cooling gels (Topp et al. 2011) and vapour coolant sprays. Moist ice is purported to be the treatment of choice for traumatic injuries (Nemet et al. 2009) despite being associated with discomfort during its application where the patient may experience aching, burning and local numbness (Hocutt 1981). They also require a freezer and at least 2 h of storage at approximately −5*°*C before use (Michlovitz 1996). Adverse effects such as skin burn and nerve damage have rarely been reported (Bleakley et al. 2004; Drez, Faust & Evans 1981). Cooling gels, which usually use menthol to elicit the cooling response, are readily available and easy to transport. However, these have been reported to result in shorter durations of decreased blood flow when compared to traditional ice application (Topp et al. 2009) and thus having a reduced effect. New ways of delivering cryotherapy often enter the market and should be tested against more traditional forms to ensure efficacy.

Physiologically, the local application of ice decreases nerve conduction velocity, tissue metabolic rate and blood flow (Algafly & George 2007). In order for inflammation to be controlled, vasoconstriction – mediated through the Rho/Rho kinase signalling pathway which is activated by changes in tissue temperature – reduces haemorrhage and oedema in the injured area (Bailey et al. 2004; 2005; Cameron 2013; Topp, Ledford & Jacks 2013), resulting in the favourable outcomes associated with cryotherapy. The cooling cuff, used in this study, consists of a textile cloth enclosing polymer granules which, when submerged in water, turn into a gel state. When applied to an area of injury, the water is gradually released from the cooling cuff, converting the gel back into the crystalline state; evaporative cooling occurs, resulting in a cooling effect on the body tissues. The purpose of this study was to compare a cooling cuff to moist ice in terms of its effect on radial artery blood flow and lumen diameter. It was hypothesised that there would be no significant difference between the two methods for either radial artery blood flow or lumen diameter.

### Research problem statement

Although many methods exist to apply cryotherapy, the high use of this therapeutic technique means that new and novel ways are always being developed to aid in the ease of its application. The cooling cuff, utilised in this study, is commercially available; yet it has not been tested against traditional moist ice therapy to determine if its effect on radial artery lumen diameter and blood flow is comparable.

### Aim of the study

This study aimed to determine the effect of a moist ice pack compared to a commercially available cooling cuff on radial artery blood flow (cm.s^−1^) and radial artery lumen diameter (mm).

## Methods

### Study population

The study population consisted of individuals residing within the eThekwini Municipality who were recruited through advertisements placed in local shopping centres, universities and sporting facilities. A total of 50 respondents were invited to an appointment at an institutional chiropractic clinic to undergo a case history and a physical and orthopaedic examination of their upper extremity to assess their eligibility to partake in the study. Prior to being examined by one of the researchers who was a student registered for a Master’s Degree in Chiropractic (J.G.), participants gave informed consent following a verbal and written explanation of the study. Participants were required to have met the study inclusion and exclusion criteria (Box 1) to be enrolled in the study. The age range of 18 and 45 years of age was selected to exclude minors who are considered a vulnerable population for research (Robinson et al. 2008) and those who could potentially have age-related changes to their skin and cardiovascular system (Sharma & Kandhpur 2009). The study was assessing blood flow in the radial artery and thus requiring that the participants were free from any diseases like hypertension, diabetes or vascular abnormalities, or habits such as smoking that could potentially affect their cardiovascular system. They were also required to not have had or currently suffered with pain in the neck or the upper extremity, as that could have affected their response to the intervention.

Box 1 Inclusion and exclusion criteria for the study.

**Inclusion criteria:**

1. 18–45 years of age
2. Asymptomatic for pain or injury to the upper limb or neck region
3. No history of trauma or surgery to the neck and/or upper limb

**Exclusion criteria:**

1. History of smoking or current smoker
2. Reported history of cardiovascular and/or peripheral vascular disease
3. Currently suffering with diabetes
4. Any cardiovascular abnormalities detected during the physical examination, for example, hypertension and peripheral vascular disease

**FIGURE 1:**
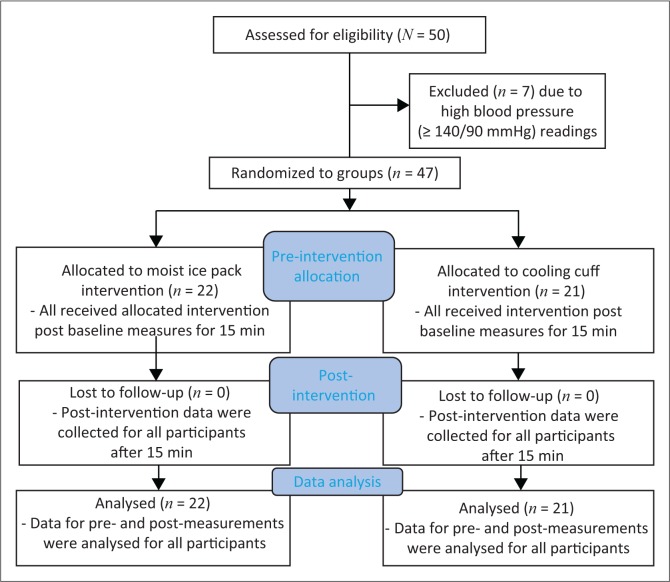
Flow diagram of participant recruitment and procedures.

Seven volunteers were excluded because of high blood pressure (≥ 140/90 mmHg), as seen in Figure 1. Those meeting the study criteria (*n* = 43), whose characteristics are tabulated in Table 1, were scheduled at their convenience within five days for an appointment at an institutional radiographic clinic where they would then participate in the data collection. Participants were asked to avoid alcohol, caffeine and physical exercise for 24 h prior to data collection to ensure that any changes, seen in radial artery blood flow, were related to the interventions and not from confounding factors. The study was approved by an institutional ethics review board.

**TABLE 1:** Characteristics of the participants per group.

Characteristics	Moist ice		SD	Cooling cuff		SD
	Number	Mean		Number	Mean	
**Gender**						
Female	6	-	-	12	-	-
Male	16	-	-	9	-	-
**Age**	-	26.59	5.94	-	28.62	6.70
Height	-	174.82	10.75	-	174.71	7.94
Weight	-	84.91	21.53	-	80.95	20.22
Body mass index	-	27.41	4.49	-	26.39	5.67
**Blood pressure^a^**						
Systolic	-	119.45	10.21	-	117.57	8.79
Diastolic	-	75.45	8.03	-	73.48	6.73

a , mm Hg; SD, standard deviation.

### Procedure

When the participants arrived at the radiographic clinic, all agreed that they had abstained from alcohol, caffeine and physical exercise for 24 h prior to the appointment. They were reminded that, by participating, they would be receiving one of two types of cold therapy to their forearm and that they may experience a burning or numb sensation during the procedure; however, once the cryotherapy was removed, the sensation would dissipate and that there would be no long-lasting effects from the application. The participant then randomly selected a piece of paper out of a bag - if they chose one they were allocated to the moist ice group and if they selected two they were part of the cooling cuff intervention. All participants arrived for the follow-up consultation as detailed in Figure 1.

Participants were required to remove any clothing covering the area below the elbow and were then instructed to relax for 15 min in a chair next to the examination table with their right side closest to the table. This allowed the participant time to acclimatise to the ultrasound room where the temperature was set to 22–23°C (De Oliveira Guirro et al. 2017). The participant was introduced to the qualified ultrasonographer who conducted all the ultrasonographic examinations and had more than 28 years of experience. The participant was then requested to place their right arm on the table, flexed to 90° at the elbow with their hand rotated at the wrist into a true lateral position with the thumb side up. All measurements were taken by the same ultrasonographer, using the same Siemens Acuson x300 Premium Edition diagnostic ultrasound machine with a 9-MHz to 13-MHz multi-frequency broadband linear transducer. The ultrasound machine was calibrated prior to the study. The ultrasonographer applied a coupling medium (generally known as the ultrasound gel) to the transducer surface to eliminate air between the face of the transducer and the skin to maximise skin contact. Diagnostic ultrasound has been reported to be accurate for measuring blood flow and, when determining cross-sectional areas of arteries, it is recommended that several measures are taken and then averaged to obtain a reading to reduce random errors (Gill 1985), as was performed in this study. Doppler ultrasound has been found to have good to very good reliability for measuring the mean and maximum blood flow velocity in peripheral arteries with intra-class correlation coefficients ranging between 0.501 and 0.866 with a standard error of measurement between 0.81 cm/s and 9.45 cm/s (De Oliveira Guirro et al. 2017).

The coupling medium, used in this study, was stored in the ultrasound room for the duration of the study. The ultrasound examination was conducted, using the B Mode and Color Flow to examine the radial artery from the elbow to the wrist joint to exclude any intraluminal filling defects. Radial artery lumen diameter (mm) was measured, using the anterior–posterior diameter measurements of the radial artery taken at the anatomical snuff box in the longitudinal plane. Three measurements were taken at this same site in the same plane to ensure reliability and reproducibility. The normal diameter of the radial artery ranges between 2.3 mm and 5 mm (Ashraf et al. 2010; Huzjan et al. 2004).

Radial artery blood flow (cm.s^−1^) was obtained by using triplex imaging which is B Mode, Color Flow and Pulse-Wave Doppler. The vessel was initially interrogated in B Mode, and Color Flow was then applied and pulse-wave Doppler was superimposed on the images for spectral analysis. The peak systolic velocities were recorded and, as in B Mode, three sets of values were obtained and recorded as well as the normal triphasic pattern of the radial artery. The accepted normal peak systolic value of the radial artery is 69 cm/s (Huzjan et al. 2004). After baseline measures were obtained, the intervention – either the moist ice pack or the novel cooling cuff – was applied. The moist ice intervention consisted of 0.6 kg of ice cubes taken from a freezer (temperature −18°C), weighed with the same kitchen scale and wrapped in a damp cloth which was then secured with an elastic band. The ice pack was then applied to the participant’s ventral right forearm two finger breadths distal to the subjects elbow crease and covered with a light towel which was placed over the moist ice pack and lightly tucked underneath the participant’s forearm to prevent the ice pack from falling. Ice cubes were used over crushed ice, as they have been found to be superior in reducing surface and intramuscular temperatures (Dykstra et al. 2009). The weight of 0.6 kg was utilised as Janwantanakul (2009) found that this weight was superior to 0.3 kg or 0.8 kg, resulting in tissue temperature changes. The cooling cuff (Recoolx^®^ Sievers, Bramsche, Germany), utilised in this study, was 33 cm in length, 16 cm in width and was less than 1 cm thick which became approximately 2 cm–4 cm thick once ready for use. In order to activate the cooling cuff, it was immersed in water for 20 min. This triggered the polymer crystals embedded within the textile fabric to attract water (Öko Tex 100, high Tech™ Micro), and thereby activating its cooling properties. This was performed while the participant was acclimatising to the ultrasound room. The cuff was then wrapped around the participants’ forearm, ensuring that that it was two finger breadths distal to the participants elbow crease, ensuring that the polymer crystal belly was over the participants forearm. It was then secured with the velcro strap that was attached to the cooling cuff.

Once the cryotherapy technique was in place, it was administered for 15 min which was timed by using a stopwatch. In order for ice to retain sufficient cooling temperatures, it has been recommended that it is applied for 15–20 min (Bleakley, Glasgow & Webb 2012; Michlovitz 1996; Topp et al. 2013). Thereafter, the participant’s wrist was rotated back into the true lateral position, thumb side up to take the radial artery lumen diameter and blood flow measures in the same manner as for the baseline measures. Cryotherapy application has been reported to result in reflex increases in blood flow for application longer than 15 min (Cameron 1999). Figure 1 provides details of the research procedure. When the post-measures for radial artery diameter and blood flow (three each) had been taken and recorded, cryotherapy was removed and the participant was asked to comment if they had experienced any discomfort during the application of the cryotherapy technique with either a yes or no answer. Their response was recorded on the data collection sheet. Thereafter, they were requested to redress and were thanked for their time and were allowed to leave. During baseline and post-measures, the same researcher (J.G.) recorded the three values obtained from the ultrasonographic examination per participant at the time of the examination, each for radial artery diameter (mm) and blood flow (cm.s^−1^) on a data collection sheet. This sheet was compiled to record the measures for each participant for ease during data capturing. All ultrasound images were stored in the hard drive of the ultrasound unit for the duration of the study; on completion of the study, they were removed.

### Statistical analysis

Statistical package for the social sciences (SPSS Base for Windows, version 20, IBM Corp. released 2010, IBM SPSS Statistics for Windows, Version 20.0, Armonk, NY) was utilised by a statistician from the University of Stellenbosch to analyse the data. The three values for radial artery diameter (mm) and for blood flow (cm.s^−1^) were averaged to get one reading per participant for each for radial artery diameter (mm) and blood flow (cm.s^−1^). Baseline differences between the groups were assessed, using Pearson’s chi-square tests for categorical variables and independent sample student’s *t*-tests for numerical data. Paired student’s *t*-tests were used to determine changes in radial artery blood flow and lumen diameter within the groups, while repeated measures analysis of variance assessed changes over the study period between the two groups. Results were considered significant at *p* < 0.05. Percentage changes in radial blood flow and diameter were calculated to determine clinical significance.

### Ethical considerations

All participants were informed, verbally and through a written letter of information, about the study and that they had the right to withdraw at any time without any repercussions. Participants’ confidentiality and anonymity were maintained by using coding. All research data were placed in the participants’ file that was stored in the chiropractic clinic during the course of the study. The ultrasound images were stored on the ultrasound machine in the Department of Radiography at the Durban University of Technology which were then removed from the machine on completion of the study. All research data were then stored in the chiropractic programme at the Durban University of Technology.

## Results

**FIGURE 2:**
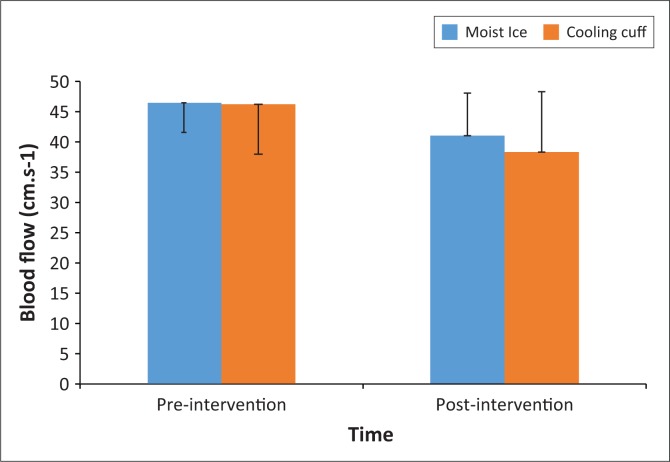
The mean and standard deviation of pre- and post-measurements for radial artery blood flow (cm.s^−1^) per group.

**FIGURE 3:**
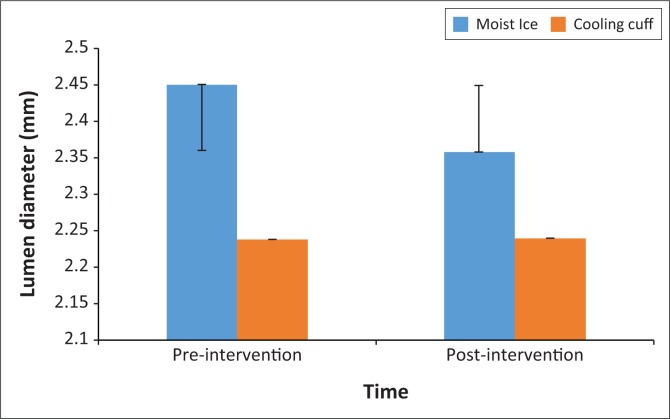
The mean and standard deviation of pre- and post-measurements for radial artery lumen diameter (mm) per group.

There were no differences between the two groups in terms of age (*p* = 0.15), body mass index (*p* = 0.51) and blood pressure (systolic *p* = 0.52; diastolic *p* = 0.67). There was a higher number of male participants in the moist ice group (*p* = 0.05; Pearson’s chi-square = 3.94, Table 1). Gender was used as a covariate in the analysis to correct for this imbalance. No differences were found between the two groups in terms of radial artery lumen diameter (*p* = 0.18) and blood flow (*p* = 0.94) at baseline. Intra-group analysis showed that both groups had a decrease in arterial blood flow (moist ice *p* < 0.001; cooling cuff *p* < 0.001; Figure 2) with neither group showing a significant change in radial artery diameter (moist ice *p* = 0.16; cooling cuff *p* = 1.00; Figure 3) over time. The moist ice and cooling cuff groups had a mean reduction in blood flow of 11.1% and 17.7%, respectively. When the groups were compared, no significant differences were found between the groups for radial arterial blood flow (*p* = 0.81; Figure 2) or lumen diameter (*p* = 0.54; Figure 3). *Post hoc* power analysis showed a power of 32%, alpha 0.5. None of the participants in the cooling cuff group reported any discomfort; in contrast, several participants in the moist ice group reported that that they experienced mild discomfort towards the end of the 15 min of application which subsided shortly after the moist ice pack was removed.

## Discussion

In this prospective investigational study, a cooling cuff, which we believe has not been tested against moist ice before, was assessed for its ability to result in changes that were similar to those seen with moist ice. Both the moist ice and the cooling cuff resulted in significant decreases in radial artery blood flow with neither intervention being superior to the other. Thus, the null hypothesis for this study could not be rejected. It is well documented that ice decreases blood flow (Fiscus, Kaminski & Powers 2005; Ho et al. 1994; Topp et al. 2011; 2013; Weston et al. 1994) and it is encouraging that, in this study, the cooling cuff group showed similar reductions in blood flow to the moist ice group.

Changes in blood flow, following the cryotherapy used in this study, can be attributed to the mechanism of heat conduction – the body tissues lose heat to the cooling device in order for the tissues to cool (Merrick, Jutte & Smith 2003; Swenson, Swärd & Karlsson 1996) and not that the cooling device transfers cold to the tissues. During this process, the cooling device is warmed and a phase change occurs which results in superior heat transfer (Kennet et al. 2007; Smith et al. 1993) and tissue cooling. The ultrasound gel, used in this study, was stored in the ultrasound room where the temperature was controlled at 22°C–23°C. It was applied to the ultrasound head in both groups in a similar manner. It has been reported that, initially, the gel can be perceived as cold which may have affected blood flow in the area. However, it was applied to both groups in the same manner and studies show that, in order to affect blood flow, cryotherapy techniques are required to be applied for timeframes longer than 10 min (Bleakley et al. 2012). The moist ice pack, used in this study, resulted in the ice, melting and changing to water, and the towel which was wrapped around the ice, absorbed the water making it wet. Likewise, when water was lost from the polymer crystals in the cooling cuff, returning them to their crystallised form, the water made the covering textile cloth damp. Cooling devices, such as these, absorb heat through conduction and evaporation (Kennet et al. 2007; Merrick et al. 2003) and have been associated with improved tissue cooling. The findings of this study show that the cooling cuff allowed for heat transfer in a similar fashion to moist ice, resulting in a decrease in radial artery blood flow. Although attempts were made to have the moist ice application be similar in size to the cooling cuff, a small difference still existed. Janwantanakul (2009) found that, when comparing ice packs of different sizes, no significant differences were observed at the ice pack skin interface, resulting in this having a negligible effect on the effect of the cryotherapy application. However, the degree to which the tissues respond to the cryotherapy technique may have been different; yet, no difference was found between their effect on blood flow and lumen diameter.

The changes in radial artery blood flow were seen in isolation to changes in radial artery diameter. Blood flow changes by following cryotherapy techniques that are proposed to result in vasoconstriction as a result of the activation of the Rho/Rho kinase signalling pathway which results in a translocation of the two adrenoceptor to the plasma membrane and alters the sensitivity of the cutaneous vessels to calcium contraction (Bailey et al. 2004). This mechanism has been reported to affect cutaneous vessels and, as seen in this study, the effect on a medium-sized artery such as the radial artery was negligible. It is plausible that the measurement tool was not sensitive enough to detect changes in radial artery diameter or that the duration of ice application was insufficient. Prolonged exposure to ice therapy has been associated with adverse reactions in as little as 20–30 min of application (Bleakley et al. 2012; 2004), making prolonged applications risky. Topp et al. (2013) reported similar findings when ice, a menthol gel, a combination of the two and a control group were compared for changes in radial artery diameter and blood flow at 5, 10, 15 and 20 min where no changes in diameter occurred, but blood flow changes were found.

In our study, final measurements were taken after 15 min of cryotherapy application. Michlovitz (1996) and Topp et al. (2013) have recommended that between 15 and 20 min is sufficient for cold packs to retain their cooling temperature. Topp et al. (2013) found that after 15 and 20 min of crushed ice application changes in radial artery blood flow occurred, as was seen in this study for both the cooling cuff and moist ice pack groups. The hunting reaction occurs when temperatures drop below 10°C which can occur after ice applications of longer than 15 min, leading to reflex vasodilation and an increase in blood flow to the area concerned (Cameron 2013). In an attempt to prevent this, the measurements were taken at 15 min. If ice is applied to the body for prolonged periods, it may lead to cooling below critical temperatures (15°C) resulting in soft tissue damage and skin burns (Cameron 2013; Swenson et al. 1996). A recent review found no incidence of skin burns associated with ice application (Bleakley & Hopkins 2010). The disadvantage of using ice packs is that they require at least 2 h of freezing at approximately −5°C before use and once the ice melts, refreezing is required. In this study, several participants who received moist ice reported mild discomfort. Similar findings have been reported by Topp et al. (2013). The benefit of the cooling cuff is that it is comfortable to wear and can be worn during cryotherapy application and, thereby, limiting interference with activities of daily living. It is reusable and does not require refrigeration or result in excessive wetness often associated with moist ice. These qualities may make it preferable as a cryotherapy application for people with musculoskeletal pain or injuries where they may select the cooling cuff over conventional icing therapies. In addition, these findings may be of interest to manual therapists, sports physicians, coaching staff and doctors who treat patients with musculoskeletal pain or injury who may elect to use or recommend the cooling cuff to their patients.

## Limitations

The cooling cuff had a velcro strap that allowed it to be secured to the participants arm, whereas the moist ice pack was held in place by a light towel, the effect of this on the outcome is unknown and it is recommended that future studies account for this variable. The sample size, utilised in this study was small and thus a larger sample size might yield different results. The study relied on the honesty of the participants, as they did not perform any exercise or consume any stimulants prior to participating in the study, If they had, their blood flow may have been affected which could have skewed the results.

## Recommendations

Future studies should be conducted to further investigate the use of the cooling cuff in participants with acute musculoskeletal pain, compared to moist ice and other types of cryotherapy to determine its effectiveness. Repeating this study with larger sample size will allow for generalisability of the results. The manufacturer advocates that the cooling cuff can safely be applied for longer durations. The effect of prolonged application requires further investigation.

## Contribution to the field

A study of this nature is beneficial to healthcare practitioners, sportsmen and women, and the public who use cryotherapy as part of their treatment regime, as it highlights a new and innovative method of cryotherapy application. This study provides evidence that the cooling cuff does decrease arterial blood flow similar to that of traditional moist ice and thus indicating that the potential for it to provide the same physiological benefits of moist ice exists.

## Conclusion

The purpose of this study was to determine if the cooling cuff would result in similar changes in radial blood flow and lumen diameter as moist ice, with the aim that clinicians could use the cuff with assurance that it was resulting in physiological changes similar to those experienced with ice therapy. The results of the study support the hypothesis. In addition, the cooling cuff was comfortable with no test subjects reporting any adverse reactions, while several participants in the moist ice group reported mild discomfort. These findings indicate that the cooling cuff can be a favourable form of cryotherapy that does not require freezing, making it readily available. It is also possible to wrap around the injured area, resulting in it being comfortable to wear.

## References

[CIT0001] Algafly A.A. & George K.P., 2007, ‘The effect of cryotherapy on nerve conduction velocity, pain threshold and pain tolerance’, *The British Journal of Sports Medicine* 41(6), 365–369. 10.1136/bjsm.2006.031237 17224445 PMC2465313

[CIT0002] Ashraf T., Panhwar Z., Habib S., Memon M.A., Shamsi F. & Arif J., 2010, ‘Size of radial and ulnar artery in local population’, *Journal of Pakistan Medical Association* 60(10), 817–819. 21381609

[CIT0003] Bailey S.R., Eid A.H., Mitra S., Flavahan S. & Flavahan N.A., 2004, ‘Rho kinase mediates cold-induced constriction of cutaneous arteries’, *Circulation Research* 94(10), 1367–1374. 10.1161/01.RES.0000128407.45014.58 15087420

[CIT0004] Bailey S.R., Mitra S., Flavahan S. & Flavahan N.A., 2005, ‘Reactive oxygen species from smooth muscle mitochondria initiate cold-induced constriction of cutaneous arteries’, *American Journal of Physiology Heart and Circulatory Physiology* 289, H243–H250. 10.1152/ajpheart.01305.2004 15764673

[CIT0005] Bleakley C., Mcdonough S. & Mac Auley D., 2004, ‘The use of ice in the treatment of acute soft-tissue injury: A systematic review of randomized controlled trials’, *American Journal of Sports Medicine* 32(1), 251–261. 10.1177/0363546503260757 14754753

[CIT0006] Bleakley C.M., Glasgow P. & Webb M.J., 2012, ‘Cooling an acute muscle injury: Can basic scientific theory translate into the clinical setting?’, *British Journal of Sports Medicine* 46(4), 296–298. 10.1136/bjsm.2011.086116 21677317

[CIT0007] Bleakley C.M. & Hopkins J.T., 2010, ‘Is it possible to achieve optimal levels of tissue cooling in cryotherapy?’, *Physical Therapy Reviews* 15(4), 344–350. 10.1179/174328810X12786297204873

[CIT0008] Cameron M.H., 1999, *Physical agents in rehabilitation: From research to practice*, W.B. Saunders Company, Philadelphia, PA.

[CIT0009] Cameron M.H., 2013, *Physical agents in rehabilitation: From research to practice*, W.B. Saunders Company, Philadelphia, PA.

[CIT0010] De Oliveira Guirro E.C., de Paula Marcondes Ferreira Leite G., Dibai-Filho A.V., de Souza Borges N.C. & de Jesus Guirro R.R., 2017, ‘Intra- and inter-rater reliability of peripheral arterial blood flow velocity by means of Doppler ultrasound’, *Journal of Manipulative and Physiological Therapeutics* 40(4), 236–240. 10.1016/j.jmpt.2017.02.007 28390709

[CIT0011] Drez D., Faust D.C. & Evans J.P., 1981, ‘Cryotherapy and nerve palsy’, *The American Journal of Sports Medicine* 9(4), 256–257. 10.1177/036354658100900414 7258468

[CIT0012] Dykstra J.H., Hill H.M., Miller M.G., Cheatham C.C., Michael T.J. & Baker R.J., 2009, ‘Comparisons of cubed ice, crushed ice, and wetted ice on intramuscular and surface temperature changes’, *Journal of Athletic Training* 44(2), 136–141. 10.4085/1062-6050-44.2.136 19295957 PMC2657028

[CIT0013] Enwemka C.S., Allen C., Avila P., Bina J., Konrade J. & Munns S., 2002, ‘Soft tissue thermodynamics before, during, and after cold pack therapy’, *Medicine and Science in Sports and Exercise* 34(1), 45–50. 10.1097/00005768-200201000-00008 11782646

[CIT0014] Fiscus K.A., Kaminski T.W. & Powers M.E., 2005, ‘Changes in lower-leg blood flow during warm-, cold- and contrast-water therapy’, *Archives of Physical Medicine and Rehabilitation* 86(7), 1404–1410. 10.1016/j.apmr.2004.11.046 16003672

[CIT0015] Gill R.W., 1985, ‘Measurement of blood flow by ultrasound: Accuracy and sources of error’, *Ultrasound in Medicine and Biology*. 11(4), 625–641. 10.1016/0301-5629(85)90035-3 2931884

[CIT0016] Ho S.S.W., Marc C.N., Kagawa R.W. & Richardson A.B., 1994, ‘The effects of ice on blood flow and bone metabolism in knees’, *American Journal of Sports Medicine* 22(4), 537–540. 10.1177/036354659402200417 7943521

[CIT0017] Hocutt J.E., 1981, ‘Cryotherapy’, *American Academy of Family Physicians* 23, 141–144.

[CIT0018] Huzjan R., Brkljacic B., Brkljacic-Delic D., Biocina B. & Sutlic Z., 2004, ‘BMode and Colour Doppler Ultrasound of the forearm arteries in the preoperative screening prior to coronary artery bypass grafting’, *Collegium Antropologicum* 28, 235–241. 15571096

[CIT0019] Janwantanakul P., 2009, ‘The effect of quantity of ice and size of contact area on ice pack/skin interface temperature’, *Physiotherapy* 95, 120–125. 10.1016/j.physio.2009.01.004 19627693

[CIT0020] Kennet J., Hardaker N., Hobbs S. & Selfe J., 2007, ‘Cooling efficiency of 4 common cryotherapeutic agents’, *Journal of Athletic Training* 42(3), 343–348. 18059988 PMC1978470

[CIT0021] Merrick M.A., Jutte L.S. & Smith M.E., 2003, ‘Cold modalities with different thermodynamic properties produce different surface and intramuscular temperatures’, *Journal of Athletic Training* 38(1), 28–33. 12937469 PMC155508

[CIT0022] Michlovitz L.S., 1996, *Thermal agents in rehabilitation*, F.A. Davis Company, lPhiladelphia, PA.

[CIT0023] Nadler S.F., Weingand K. & Kruse R.J., 2004, ‘The physiologic basis and clinical applications of cryotherapy and thermotherapy for the pain practitioner’, *Pain Physician* 7, 395–399. 16858479

[CIT0024] Nemet D., Meckel Y., Bar-sela S., Zaldivar F., Cooper D.M. & Eliakim A., 2009, ‘Effect of local cold-pack application on systemic anabolic and inflammatory response to sprint-interval training: A prospective comparative trial’, *European Journal of Applied Physiology* 107, 411–417. 10.1007/s00421-009-1138-y 19652995 PMC2762537

[CIT0025] Robinson J.A., Human S., Boshoff A., Smith B.S. & Carnelly M., 2008, *Introduction to South African family law*, 3rd edn., Butterworth, London.

[CIT0026] Sharma V.K. & Kandhpur S., 2009, ‘Guidelines for cryotherapy’, *Indian Journal of Dermatology, Venereology and Leprology* 75(2), 90–100.

[CIT0027] Smith T.L., Curl W.W., Smith B.P., Holden M.B., Wise T., Marr A. et al., 1993, ‘New skeletal muscle model for the longitudinal study of alterations in microcirculation following contusion and cryotherapy’, *Microsurgery* 14(8), 487–493. 10.1002/micr.1920140805 8271927

[CIT0028] Swenson C., Swärd L. & Karlsson J., 1996, ‘Cryotherapy in sports medicine’, *Scandinavian Journal of Medicine and Science in Sports* 6(4), 193–200. 10.1111/j.1600-0838.1996.tb00090.x 8896090

[CIT0029] Topp R., Ledford E.R. & Jacks D.E., 2013, ‘Topical menthol, ice, peripheral blood flow, and perceived discomfort’, *Journal of Athletic Training* 48(2), 220–225. 10.4085/1062-6050-48.1.19 23672386 PMC3600924

[CIT0030] Topp R., Winchester L., Mink A.M., Kaufman J.S. & Jacks D.E., 2011, ‘Comparison of the effects of ice and 3.5% menthol gel on blood flow and muscle strength of the lower arm’, *Journal of Sports Rehabilitation* 20(3), 355–366. 10.1123/jsr.20.3.355 21828387

[CIT0031] Topp R., Winchester L., Sannes S.H., Mink A.M., Kaufman J.S. & Jacks D.E., 2009, ‘A comparison of Biofreeze and ice on blood flow, pain and muscle function (Abstract)’, viewed 31 October 2012, from http://www.thera-bandacademy.com/resource/x-showResource.aspx?id=3445

[CIT0032] Weston M., Taber C., Casagranda L. & Cornwell M., 1994, ‘Changes in local blood volume during cold gel pack application to traumatized ankle’, *Journal of Orthopaedic and Sports Physical Therapy* 19(4), 197–199. 10.2519/jospt.1994.19.4.197 8173566

